# Localization of deformed wing virus (DWV) in the brains of the honeybee, *Apis mellifera *Linnaeus

**DOI:** 10.1186/1743-422X-6-182

**Published:** 2009-10-30

**Authors:** Karan S Shah, Elizabeth C Evans, Marie C Pizzorno

**Affiliations:** 1 Department of Biology, Bucknell University, Lewisburg, PA 17837, USA; 2 Animal Behavior Program, Bucknell University, Lewisburg, PA 17837, USA; 3 Cell Biology and Biochemistry Program, Bucknell University, Lewisburg, PA 17837, USA

## Abstract

**Background:**

Deformed wing virus (DWV) is a positive-strand RNA virus that infects European honeybees (*Apis mellifera *L.) and has been isolated from the brains of aggressive bees in Japan. DWV is known to be transmitted both vertically and horizontally between bees in a colony and can lead to both symptomatic and asymptomatic infections in bees. In environmentally stressful conditions, DWV can contribute to the demise of a honeybee colony. The purpose of the current study is to identify regions within the brains of honeybees where DWV replicates using *in-situ *hybridization.

**Results:**

*In-situ *hybridizations were conducted with both sense and antisense probes on the brains of honeybees that were positive for DWV as measured by real-time RT-PCR. The visual neuropils demonstrated detectable levels of the DWV positive-strand genome. The mushroom bodies and antenna lobe neuropils also showed the presence of the viral genome. Weaker staining with the sense probe in the same regions demonstrates that the antigenome is also present and that the virus is actively replicating in these regions of the brain.

**Conclusion:**

These results demonstrate that in bees infected with DWV the virus is replicating in critical regions of the brain, including the neuropils responsible for vision and olfaction. Therefore DWV infection of the brain could adversely affect critical sensory functions and alter normal bee behavior.

## Background

European honeybees (*Apis mellifera *L.) play a crucial role in agricultural industries by pollinating crops [1,2]. Unlike other pollinators, these bees are generalist foragers that readily visit multiple types of plants; they serve as an alternative to the species-specific pollinators [3]. In addition, honeybees are flower-constant, meaning that they usually restrict their visits to flowers of the same species during foraging flights and bypass valuable, alternative food sources [4]. This behavioral trait is thought to increase pollination efficiency of these pollinators compared to others.

There have been at least 16 viruses, primarily of the picorna-like group of positive-strand RNA viruses, known to infect honeybees [5]. One commonly detected honeybee virus is the deformed wing virus (DWV), which belongs to the *Iflavirus *genus, a group of viruses distantly related to human picornaviruses, like polio and rhinovirus [6,7]. DWV was first isolated from honeybees in the 1980s in Japan and is now found in all parts of the world where *Varroa *mites are found [5,8,9]. *Varroa destructor *is a mite that parasitizes immature and adult bees by feeding on them and has the ability to serve as a vector to transmit the virus horizontally [10-13]. A high density of mites in a colony suppresses the immune function of the bees by reducing the transcription of antimicrobial peptides and immune related enzymes [14,15]. The reduced levels of these immune related enzymes appears to exacerbate DWV infection and can lead to the demise of the colony [15].

Even in the absence of the mites, DWV can be transmitted vertically through infected eggs and sperm [16-18]. Typical symptoms exhibited by severely infected bees are crumpled wings, bloated abdomens, paralysis, learning deficits, and a drastically shortened life span and there is a direct correlation between the viral titer and the symptoms displayed [10]. Symptoms of the viral infection are most prevalent during stressful environmental conditions, leading to reduced performance in infected colonies [19-21]. Therefore as the number of infected worker bees increases, the colony is less likely to survive.

DWV is a positive-strand RNA virus that produces a 30-nm icosahedral particle composed of three major structural proteins [6]. As a member of the *Iflavirus *genus, DWV has a typical picornavirus genome organization consisting of a single open reading frame flanked by 1144 nt 5' and 317 nt 3' nontranslated regions, which contain putative replication and translational control elements [6,22]. The viral RNA is presumed to be polyadenylated and the structural proteins are N-terminal to the non-structural proteins [6]. DWV genome sequences are highly conserved in most parts of the world [9] and have a very close sequence homology to two other iflaviruses Kakugo virus (KV) and Varroa destructor virus 1 (VDV-1) [6,23,24]. Currently there is disagreement on whether DWV and KV are actually regional isolates of the same virus [6] or two separate species [24]. KV was first isolated from aggressive bees in Japan; however, there has been no direct correlation made between the aggressiveness of the bees and the presence of KV or DWV [25]. Specific molecular techniques have been developed to sensitively detect both DWV and KV genomes [6,23,26], however the high sequence homology between the two viruses makes it difficult to differentiate between them. While it has also been shown that DWV infection impairs associative learning [27], it has not been shown to increase aggression as has been proposed for KV [25].

In this study, we identified the presence and replication of DWV viral RNA in several areas of the brains of infected honeybees. The data presented here show that DWV localizes in the olfactory and visual regions, which may alter the normal behavior of the bees and contribute to the demise of the colony.

## Results

### Real Time PCR Results

Real-time RT-PCR was used to quantify DWV viral RNA and to identify bees that carried relatively high viral loads (Table 1). This procedure was carried out on bees collected from two different hives to determine the level of DWV RNA in the bees that were used in the in situ hybridizations. Real-time RT-PCR confirmed that bees can show highly variable levels of DWV RNA, whether they are morphologically symptomatic or not for DWV infection (Table 1). The level of DWV RNA also increases in bees collected later in the season when the bees are more likely to be symptomatic. This could be due to higher levels of mite infestation later in the season or harsher environmental conditions and reduced food sources [21]. Real-time PCR data suggest that many, but not all, of the bees in Hive D carry at least low levels of DWV RNA (data not shown). Interestingly, some asymptomatic bees had very high levels of viral RNA and the brains from these bees showed a strong hybridization with the virus probes.

**Table 1:** Number of copies of the DWV genome in tested bees using real-time RT-PCR.

**Bee Number**	**Hive**	**Date Sampled**	**Symptomatic**	**DWV copies/μg total RNA**
1	B	July 2007	-	<10^3^
2	D	July 2007	-	1 × 10^5^
3	D	July 2007	-	5 × 10^6^
4	D	Sept. 2007	-	6 × 10^6^
5	D	Sept. 2007	+	4 × 10^7^
6	D	Sept. 2007	+	5 × 10^7^

**Figure 1:**
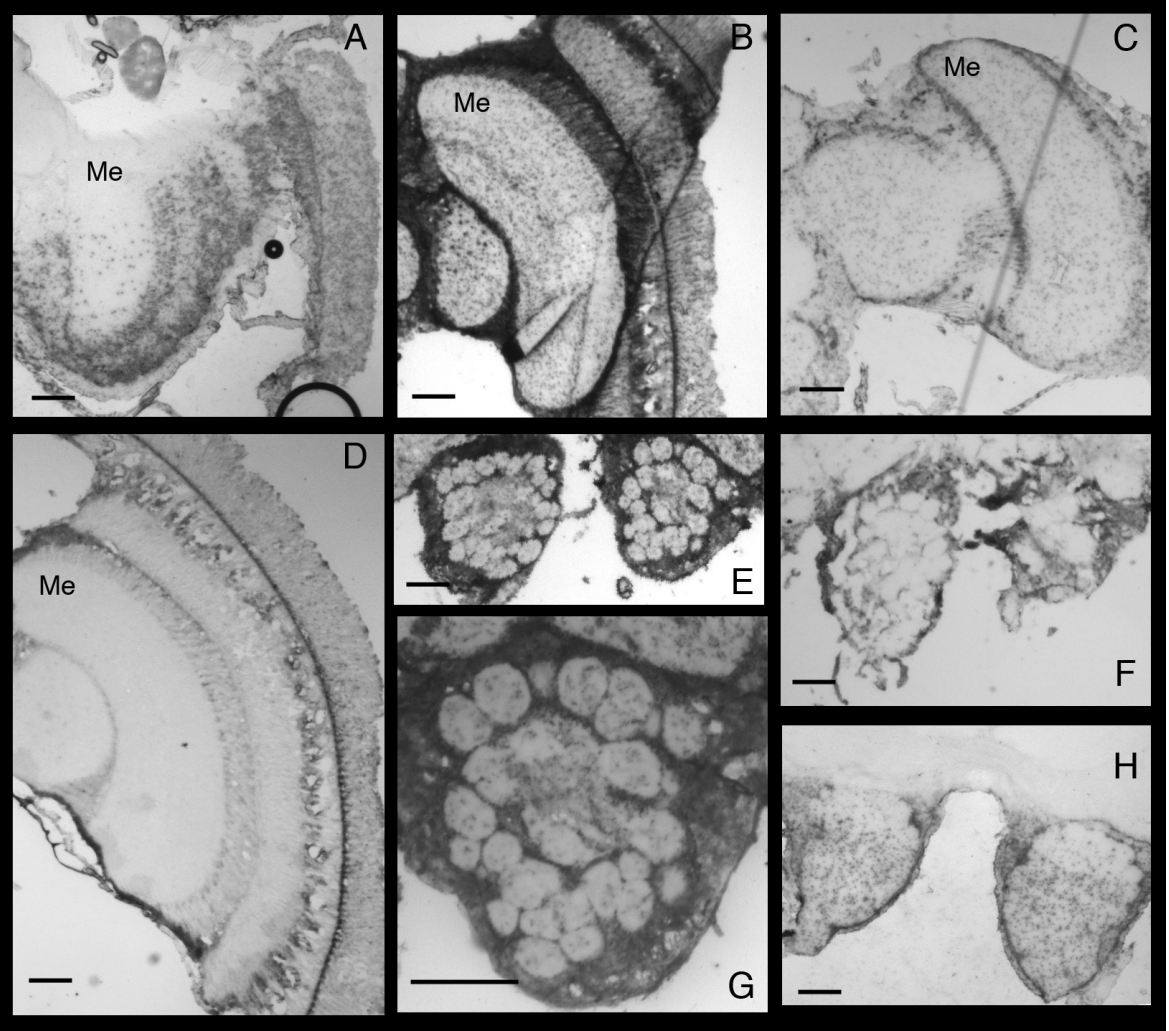
**Detection of the genome and antigenome of DWV in the optic lobe, the antenna lobe and the subesophageal ganglion of honeybee brains**. In-situ hybridization of DWV positive bee brains with antisense probes (A, B, D, E, G, H) and sense probes (C, F). A, optic lobe from bee#3 with antisense probe, 4×; B, optic lobe from bee#5 with antisense probe, 4×; C, optic lobe from bee#3 with sense probe, 4×; D, optic lobe from bee#2 with antisense probe, 4×; E, antenna lobe from bee #5 with antisense probe, 4×; F, antenna lobe from bee #5 with sense probe, 4×; G, magnification of E, 10×; H, subesophageal ganglion from bee #3 with antisense probe, 4×; bar = 100 μm. The notation Me in panels A, B, C, and D identifies the crescent shaped medulla region of the brain.

**Figure 2:**
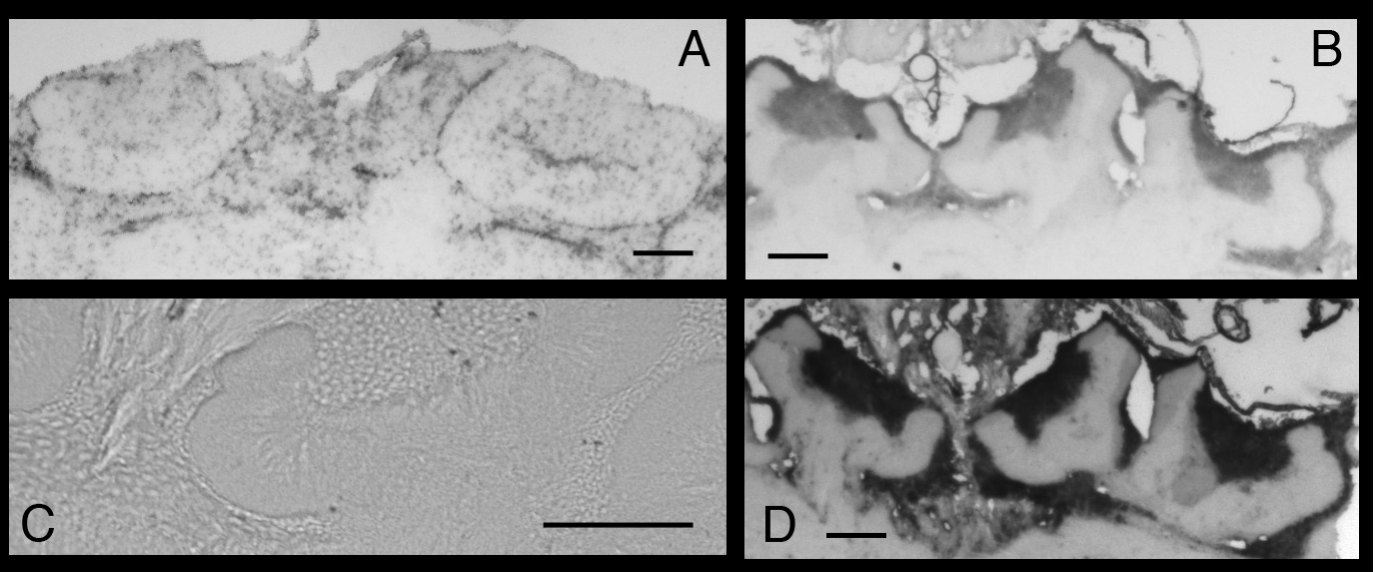
**Detection of the genome and antigenome of DWV in the mushroom bodies of honeybee brains**. In situ hybridization of mushroom bodies from DWV positive (A, B, D) and DWV negative (C) bee brains. A, bee #4 with antisense probe, 4×; B, bee #6 with sense probe, 4×, C, bee#1 with antisense probe, 4×; D, bee #6 with antisense probe, 10×; bar = 100 μm.

The number of copies of viral RNA per μg of total bee RNA was calculated for the honeybees shown in Figures 1 and 2 using real-time RT-PCR. The copy number was calculated from a standard curve of a plasmid containing the DWV amplicon. Actin was used as an internal control and the Ct numbers were within 2.0 from each other.

### In-situ Hybridization Results

We conducted in-situ hybridizations to detect the presence and localization of DWV RNA in bee brains. The positive-strand viral RNA was present in the visual neuropils, including the crescent shaped medulla, as shown from the staining pattern observed with the antisense probe (Figure 1A, B and 1D). The staining is localized in a punctate pattern throughout the medulla region of the optic lobe of the bee brain (Me, Figure 1). Two of the in-situ hybridizations (Figure 1A and 1D) were conducted on bees collected from hive D early in the summer, which showed moderate levels of the viral RNA as measured by real time PCR (Table 1) while the darker staining sections (Figure 1B, E, G) were taken from Bee #5 with higher levels of viral RNA (Table 1). To determine whether the virus is actively replicating, a second section from Bee#3 was hybridized to the sense probe (Figure 1C). The detection of the antigenome by the sense probe, while lighter staining, confirms replication of the virus in this region of the brain. The viral genome was also detected in the subesophageal ganglion found near the back of brain (Figure 1H). An additional in-situ hybridization was conducted on Bee#5 to determine if the virus infects and replicates within the antenna lobes of the brain. These results showed both viral RNA and antigenome in antenna lobes (Figure 1E-G), which are responsible for receiving and processing olfactory signals from the antenna.

Finally, because the corpora pedunculata neuropil (mushroom bodies) of insect brains is crucial for the detection and integration of external stimuli, we conducted in-situ hybridizations to determine if the virus is present in this region. In both asymptomatic Bee#2 and symptomatic Bee #6, which carries a very high level of viral RNA (Table 1), the mushrooms bodies contain viral RNA (Figure 2A and 2B). The differences in the staining pattern between the sections taken from these two bees is likely due to the differences in the level of viral RNA they carried (Table 1). In addition, Bee#6 shows the presence of the antigenomic RNA (Figure 2D). Finally, our antisense probe does not hybridize to brain sections from Bee#1 with very low levels of viral RNA (Figure 2C), as measured by real-time RT-PCR (Table 1). This finding demonstrates that the hybridization is specific and the probe does not detect any cellular RNA.

## Discussion

DWV is a positive-strand RNA virus that has a 97% sequence homology with KV [6]. Because KV was first identified in attacker bees in Japan, there has been some interest in determining if DWV/KV can cause aggressive behavior in the honeybee. Some reports do not correlate virus infection with aggression [25], while other reports suggest that they may be a link between virus infection and either aggression or learning deficits in bees [23,24,27]. Because of this link between DWV/KV infection and changes in bee behavior, we sought to determine which regions of the honeybee brain are infected with DWV/KV using in-situ hybridization.

The in-situ hybridizations results demonstrate that the DWV genomic RNA was present in high concentrations in the optic and antennal neuropils in the bee brain and in mushroom bodies. We can therefore conclude that the virus is present in the brain regions that process the bees' sensory experiences. In addition, brain sections that were hybridized to the sense probe also showed lighter staining in these same regions. This discovery demonstrates the presence of the antigenomic viral RNA and that the virus is actively replicating in these areas of infected bee brains. The lighter staining with the sense probe is expected since picorna-like viruses produce only 10% as much of the antigenomic RNA to act as the template strand for the replicating viral genomes [28]. Finally, we note that most of the viral RNA appears to be detected in a punctate pattern, which may be the cell bodies of the neurons. This finding is consistent with the known subcellular location for replication of other picornaviruses, such as polio [29]. However, we can not rule out that the punctate staining represent viral RNA replication in other cells, such as glial cells. Further staining with neuronal and glial cell markers will be needed to answer this question.

The presence of replicating virus in the optic neuropils may indicate that infected bees have impaired vision. A similar conclusion can be drawn from the evidence of high viral load in the antennal lobes: this infected neural tissue could indicate a deficit in olfactory processing. Therefore, it is possible that the virus affects the bees' flight behavior, homing performance, and perception of odorants. While future experiments will undoubtedly reveal the nature of the behavioral impairment attributable to DWV, it is at least plausible that this viral infection contributes to the bee disappearances associated with colony collapse disorder [30,31]. Recent work by Johnson et al [30] suggests that viral infections, most likely DWV since it is a common infection in many US hives, might lead to the fragmentation of rRNA within cells, affecting cellular metabolic function and leading to CCD.

It should be noted that a previous report detected the viral genome in other tissues, including the reproductive organs, in both queen and drone bees [32], but failed to detect viral RNA in the brain. We propose that the explanation for this discrepancy is that our methods, which included the use of a longer riboprobe and cryosections, produced a higher sensitivity. We also studied worker bees rather than queens or drones, which could also explain the differences in our results.

## Conclusion

We have detected both DWV genomic and antigenomic viral RNA in the brains of infected worker honeybees using in-situ hybridization. This demonstrates that DWV is not only present in the brains of bees, but is also actively replicating. Because the regions infected include those involved in sight and olfaction we predict that DWV infected bees will have difficulty with their vision or sense of smell; if either system is impaired, their ability to function normally as a productive member of the hive would be compromised.

## Methods

### Isolation of viral RNA from bees/cDNA synthesis

#### Collection of Bees

Bees were collected from the hives located in Bucknell's apiary. One of the hives, Hive D, was obtained from D. Cox-Foster, Penn State University. Bees, most likely foragers, were chosen at random upon their departure from the hive and killed by freezing at -80°C Celsius. For later experiments, both asymptomatic bees and young or newly emerged bees with crumpled wings were chosen from Hive D, which was known to be positive for DWV.

#### RNA Isolation

RNA was isolated from the abdomen and thoraxes of adult bees, whole pupae, and larvae using Trizol (Invitrogen). The bee tissues were homogenized in 800 μl of Trizol and extracted using 200 μl of chloroform. The RNA was precipitated from the aqueous fraction with 400 μl of isopropyl alcohol. The RNA pellet was washed with 75% ethanol, air dried, and then re-suspended in DEPC treated sterile water. The quality and quantity of the isolated RNA was measured using a Nanodrop spectrophotometer.

#### cDNA synthesis

In order to synthesize cDNA, 2 ug of total isolated RNA was combined with random decamers according to the procedure from RETROscript cDNA synthesis kit (Ambion).

### Standard RT-PCR and Cloning

#### PCR Primer Sequences and Condition

In order to clone the region of the DWV genome between nucleotide 8371 and 8748 to use as a probe for in-situ hybridizations, the following primer sequences were used: DWV1-F (5'-GGACTGAACCAAATCCGATGTCATCACG-3') and DWV1-R (5'-TCTCAAGTTCGGGACGCATTC-3'). PCR was conducted using Failsafe PCR kit (Epicentre) and contained 1 μl cDNA, 1 μM of forward and reverse primers, 0.5 μl of Failsafe PCR polymerase mix and 25 μl of the appropriate 2× PCR buffer.

#### PCR Product Cloning

The 378 bp PCR product was cloned into the pCR2.1 dual promoter vector using the TA cloning kit (Invitrogen) according to manufacturer's instructions. DNA sequencing was used to confirm the identity of the cloned insert. Two plasmids were used, each containing the PCR insert in opposite directions in relation to the T7 promoter. When transcribed using the T7 RNA polymerase, the pDWV-F produces RNA that is sense to the viral genome while the plasmid named pDWV-R produces RNA that is antisense to the DWV viral genome.

### Real-Time PCR

#### RT-PCR

Real Time RT-PCR was used to quantify the amount DWV RNA. Two primer sets were used to measure cellular and viral RNA (actin or DWV). Primers were designed using the Primer3 program and the specificity was confirmed using the BLAST program (NCBI). The primer sequence for actin left was (5'-GACGAGTCTGGACCATCCAT-3'), and the primer sequence for actin right was (5'-GGGATTCGGGGAATGAGTAT-3'). The DWV left primer sequence was (5'-AGCATGGGTGAAGAAATGTC-3'), and the DWV right primer sequence was (5'-ATATGAATGTGCCGCAAACA-3'), which amplifies the region between base 5288 and 5390 on the DWV genome. Each real-time PCR reaction contained 12.5 μl of 2× SYBR super mix (Bio-Rad), 1 μl of a 1:5 dilution of cDNA, and forward and reverse primers at a final concentration of 0.1 μM. A two-step real time PCR was conducted on a 96 well plate using the iCycler program (Bio-Rad) and all reactions were run in triplicate. The iCycler program was also used to determine the Ct number of each reaction. Ct numbers from the actin reactions were used to standardize the Ct numbers for DWV from the same samples. Melt curves obtained at the end of the amplification confirmed that each primer pair produced a single amplicon with a single Tm. To produce a standard curve, the 100 bp DWV amplicon was ligated into the pCR2.1 vector using a TA cloning kit (Invitrogen) and the identity of the insert confirmed by DNA sequencing. The resulting plasmid was purified from bacterial cells, quantified, and diluted from 10^2 ^to 10^10 ^copies/reaction. The standard curve reactions were run in the identical manner as the unknown samples and on the same plate. The standard curve relating Ct number to copy number was linear from 10^3 ^to 10^10 ^copies per reaction (R^2 ^= 0.99) and was used to calculate the number of viral RNA molecules per microgram of total bee RNA.

### In-Situ Hybridization

#### Production of Probe

Either the pDWV-F or pDWV-R plasmid DNA was digested with *Hin*dIII and phenol/chloroform extracted then ethanol precipitated. One μg of linear plasmid DNA was added to the Digoxiginen Labeling Mix (Roche) in-vitro transcription reaction with the T7 RNA polymerase according to manufacturer's instructions. The resulting single-stranded riboprobes of approximately 400 nt were then quantified according to the dot blot method.

#### Preparation of Bee Brains

Bee brains were dissected in DEPC treated bee saline solution and then placed in 4% paraformaldehyde/1× PBS for 2 hours. The brains were incubated in 18% sucrose/1 × PBS overnight at 4°C before being embedded into the OCT embedding medium. The embedded brains were cryosectioned at 10 μm, and mounted on FisherPlus slides. The sections were then dried overnight at 27°C and stored at -20°C until the in-situ hybridization was carried out.

#### Hybridization/Detection

The sections were fixed in 4% paraformaldehyde for 15 mins at room temperature. Sections were incubated with 10 mg/ml Proteinase K in 10 mM Tris-HCl, pH 8.0, 1 mM EDTA. Then sections were fixed again in 4% paraformaldehyde for 10 mins and then rinsed in 1 × PBS. Finally sections were dehydrated in increasing concentrations of ethanol (70%, 80%, 95%, 100%). Sections were then hybridized to the sense or antisense digoxigenin-labled riboprobes (1000 ng/ml) in a solution of 50% formamide, 10 mM Tris-HCl, pH 7.6, 200 mg/ml tRNA, 1× Denhart's solution, 10% dextran sulfate, 600 mM NaCl, 0.25% SDS, 1 mM EDTA overnight at 50°C. The sections were then washed in the following buffers at 50°C (0.2 × SSC, 2 × SSC, 2 × SSC/50% formamide, 5 × SSC). Digoxigenin-labeled probes were detected with a sheep anti-digoxigenin-alkaline phosphatase antibody according to manufacturer's instructions (Roche). Color development was done with NBT/BCIP in a buffer containing lavimisole (1 μM). Sections without any probe were used as negative controls. The sections were left in the NBT/BCIP solution for about 2 hours or until optimum staining was reached. To stop the staining, sections were incubated in 10 mM Tris-HCl, 1 mM EDTA for 10 minutes, and were rinsed briefly in ultra pure water. A coverslip was mounted on the sample using the permanent mounting agent Fluormount G. The pictures of the staining were captured using a Nikon E800 microscope and a Hamamatsu digital camera using the Simple PCI software. Adjustments of brightness and contrast of each image was done using Photoshop.

## Competing interests

The authors declare that they have no competing interests.

## Authors' contributions

MCP was responsible for producing the DWV plasmids, developing the experimental design and protocols, and assisting with data interpretation. ECE was responsible for collecting and dissecting the bee brains and assisting with data interpretation. KSS was responsible for carrying out the real-time PCR and in-situ hybridizations and researching the background information. All authors contributed to writing and editing the manuscript.
